# Visibility Is Not Equivalent to Confidence in a Low Contrast Orientation Discrimination Task

**DOI:** 10.3389/fpsyg.2016.00591

**Published:** 2016-04-26

**Authors:** Manuel Rausch, Michael Zehetleitner

**Affiliations:** ^1^Psychologie II, Catholic University of Eichstätt-IngolstadtEichstätt, Germany; ^2^Graduate School of Systemic Neurosciences, Ludwig-Maximilians-Universität MünchenMunich, Germany; ^3^General and Experimental Psychology, Ludwig-Maximilians-Universtität MünchenMunich, Germany

**Keywords:** consciousness, visual awareness, visibility, confidence, signal detection theory, metacognition

## Abstract

In several visual tasks, participants report that they feel confident about discrimination responses at a level of stimulation at which they would report not seeing the stimulus. How general and reliable is this effect? We compared subjective reports of discrimination confidence and subjective reports of visibility in an orientation discrimination task with varying stimulus contrast. Participants applied more liberal criteria for subjective reports of discrimination confidence than for visibility. While reports of discrimination confidence were more efficient in predicting trial accuracy than reports of visibility, only reports of visibility but not confidence were associated with stimulus contrast in incorrect trials. It is argued that the distinction between discrimination confidence and visibility can be reconciled with both the partial awareness hypothesis and higher order thought theory. We suggest that consciousness research would benefit from differentiating between subjective reports of visibility and confidence.

## Introduction

In the field of consciousness research, two general approaches to measuring conscious awareness are often distinguished: objective measures and subjective measures (Cheesman and Merikle, 1984; Seth et al., 2008). While objective measures have dominated cognitive psychology for the second half of the 20th century (Boring, 1953; Eriksen, 1960; Danziger, 1980), in more recent years, more and more researchers from different theoretical perspectives argued for using subjective measures in consciousness research (Dehaene and Naccache, 2001; Dienes, 2004, 2008; Ramsøy and Overgaard, 2004; Lau, 2008b; Dehaene, 2010; Timmermans and Cleeremans, 2015). A measure of awareness is considered objective if conscious experiences are ascribed to the subject based on performance in a task (Eriksen, 1960). In contrast, measures are deemed subjective if participants are required to make a report about their conscious experiences (Cheesman and Merikle, 1984). The main argument why objective measures should be accompanied by subjective measures relies on the premise that conscious experiences are not necessarily in accordance with performance in discrimination tasks (Lau, 2008b; Seth et al., 2008). On the one hand, there may be cases where discrimination performance is above chance in absence of conscious awareness. The standard example is blindsight, which is caused by lesions to primary visual cortex. These patients report to be blind in the visual hemifield contralateral to the damaged brain area. Despite their apparent blindness to stimuli presented in their visual field corresponding to the lesion, these patients are able to perform well above chance in forced-choice tasks (Weiskrantz, 1986). On the other hand, there may also be cases where conscious experience exceeds the manifest discrimination performance. For example, when participants are presented with arrays of several letters, observers report they can see all or almost all letters, but typically are able to report no more than 3 to 4 of the letters (Sperling, 1960; Block, 2011). As a consequence, it is not adequate to automatically assume an observer is aware whenever performance is above chance.

When using subjective measures to assess conscious awareness, it is crucial that the contents of subjective measures match the contents of subjective experience relevant for a specific research question (Rausch et al., 2015). At least two categories of conscious contents can be considered: sensory and non-sensory contents (Mangan, 2001): The standard examples of conscious experience are most often sensory contents, e.g., the redness of an apple. Sensory conscious contents are always tied to a specific sensory modality. It is not necessary that a sensory conscious content is in fact mediated by the corresponding sense organs; e.g., a hallucinated voice is also a sensory conscious experience. Many non-sensory experiences are related to processing quality (Winkielman et al., 2015). Examples in the context of implicit cognition are familiarity (Yonelinas, 2002), feeling-of-knowing (Nelson, 1984), and rightness. Familiarity has been in the focus of a considerable amount of memory research because familiarity was proposed as a second process in addition to recollection of details involved in recognition task (see Yonelinas, 2002, for a review). However, rightness was argued to be the most important non-sensory experience in implicit cognition (Mangan, 2001): it was characterized as at once the core feeling of positive evaluation, of coherence, of meaningfulness, and knowledge. For example, in artificial grammar tasks, participants’ intuition differentiated between correct and incorrect responses although participants denied their response was based on memory or knowledge (Dienes and Scott, 2005). In an artificial grammar task, confidence ratings imposed more liberal criteria task than subjective reports about grammar rule awareness (Wierzchoń et al., 2012).

Is there empirical evidence for non-sensory experiences in absence of visual awareness? Famous examples for dissociations between sensory and non-sensory experiences stem from conditions when the visual system is impaired: After lesions to primary visual cortex, so-called blindsight patients report to be blind in the visual field contralateral to the impaired brain area, although they are able to discriminate visual stimuli presented in their seemingly blind visual field in forced-choice tasks with remarkable accuracy (Weiskrantz, 1986). In blindsight type 2, patients report a form of awareness without content, a feeling of something happening that is qualitately different from normal seeing (Weiskrantz et al., 1995; Sahraie et al., 2002). Some blindsight type 2 patients also report a considerable degree of confidence that discrimination responses about stimuli presented in their blind hemifield were correct (Sahraie et al., 1998), and may even wager the same amount of money on judgments on stimuli in the blind as in the intact hemifield when the objective difficulty of judgments is balanced (Persaud et al., 2011). Confidence ratings and wagering normally depend on all conscious content the observer considers relevant for making a discrimination judgment (Dienes, 2008); however, when type 2 blindsight patients at the same time report that sensory conscious contents are absent, confidence, and wagering are more probably based on non-sensory conscious contents. A similar pattern as type 2 blindsight can be observed when neural activity in occipital cortex was only transiently disrupted via transcranial magnetic stimulation (TMS): Occipital TMS between 86 and 114 ms after stimulus onset suppressed visibility of the stimulus. Nevertheless, performance in orientation and color discrimination tasks was still quite accurate, and discrimination confidence was strongly correlated with performance in these tasks (Boyer et al., 2005).

However, there is an on-going controversy whether the conscious contents underlying type 2 blindsight qualify as sensory or not (Foley and Kentridge, 2015). On the one hand, the residual perceptual abilities of blindsight patients may indicate that the conscious experiences of blindsight type 2 patients is too fragmented to qualify as visual awareness (Kentridge, 2015). On the other hand, it was argued that type 2 blindsight should be considered as visual (and thus sensory) because it meets objective criteria for vision. The physical stimuli that caused the experience are photons and the sense organs that mediate the experience are the same as in normal vision, namely photons and the eyes (Foley, 2014). According to this view, blindsight patients report non-sensory experience without sensory experience because their reports are not always reliable (Foley, 2014). Indeed, there is evidence that at least some blindsight patients do report some visual experience when carefully queried (Overgaard et al., 2008; Ffytche and Zeki, 2011). Similar to blindsight type 2, there is a case of an achromatic patient, who reports color blindness after occipital brain damage but performs well in color discrimination tasks, and his confidence in being correct in the task strongly correlates with task performance (Carota and Calabrese, 2013). Again, it is controversial whether the performance of achromatic patients occurs in complete absence of color experiences or is mediated by residual conscious contents of color (Heywood et al., 1998).

Is there empirical evidence for non-sensory experiences in absence of visual awareness in observers without neurological impairment? When observers were asked to categorize their experience during a psychophysical experiment, the majority of participants ended up with four categories. While the first category indicated the absence of all experience, the second was described as a non-sensory experience, “a feeling of something being shown, not characterized by any content” (Ramsøy and Overgaard, 2004, p. 12). The third and the fourth category involve both sensory experiences of the stimulus as well as confidence in the discrimination judgment. This categorization developed by participants was named *Perceptual Awareness Scale* (PAS). Although many researchers have referred to the PAS as a direct measure of visual experience, it is important to note that the PAS assesses both sensory as well as non-sensory conscious contents. Other studies have directly compared subjective reports of visibility against a measure indicative of non-sensory experience. In a masked digit discrimination task, participants were able to detect their own discrimination errors in trials where they also reported the masked target digit was not visible (Charles et al., 2013, 2014). Similarly, participants were more liberal to report confidence in being correct in the discrimination task than to report visibility in a masked localization task (Schlagbauer et al., 2012), as well as a masked orientation discrimination task, a masked shape discrimination task, and a random dot motion discrimination task (Zehetleitner and Rausch, 2013). In the latter three experiments, discrimination confidence consistently outperformed subjective reports of visibility in predicting discrimination accuracy (Zehetleitner and Rausch, 2013; Rausch et al., 2015). Two other studies found different relationships between confidence ratings and the PAS: In a masked object classification task, PAS ratings imposed more liberal minimal criteria than confidence ratings, and the PAS outperformed confidence in predicting accuracy (Sandberg et al., 2010). In a masked face discrimination task, confidence was only more predictive of trial accuracy when the confidence rating was presented before the discrimination judgment; the relationship reversed when the order of task and rating was exchanged (Wierzchoń et al., 2014). As the PAS is explicitly intended to measure both visual experiences as well as conscious contents that qualify as non-sensory (Ramsøy and Overgaard, 2004), the PAS might outperform other scales in predicting accuracy because it assesses both visual experience as well as feelings of confidence. However, there may be various other explanations for the discrepant results; specifically, it was proposed that the PAS is specifically suited to measure awareness of simple visual stimuli (Sandberg et al., 2013). As a consequence, the question arises whether the effect of confidence and visibility as contents of subjective reports generalizes to other stimulus material. In addition, considering both doubts about the reliability of reports in type 2 blindsight as well as the inconsistent results of previous studies, it appears necessary to test how robust the effect of confidence vs. visibility occurs.

The present study was conducted to test if the effect of confidence and visibility as content of subjective reports on criteria and the relation between reports and accuracy is replicable and generalizes to a low contrast discrimination task. To ensure that subjective reports of visibility and discrimination confidence were based on the same amount of sensory evidence and the same discrimination bias, we required participants to report visibility as well as confidence in each single trial. Collecting two subjective reports per single trial may have two disadvantages. First, the first subjective report might influence the following second report. In addition, the second subjective report may increase the task demand, interfering with observers’ ability to monitor their own experiences or performance. However, as we balance the order of the visibility and confidence ratings across participants, potential effects of order or task demand should be orthogonal to the effect of interest for the purpose of the present study. We provided participants with feedback about incorrect discrimination judgments after their subjective reports to maintain consistency with existing studies on discrimination confidence (Pleskac and Busemeyer, 2010; Moran et al., 2015) and our own previous study (Zehetleitner and Rausch, 2013). Moreover, to investigate the relation to the quality of stimulation, we varied the contrast of the target stimulus. We compared five statistics between visibility and confidence: average reports, the probability of reporting confidence and visibility conditioned on the other report within the same trial, meta-*d*_a_ as measure of the degree to which subjective reports differentiate between correct and incorrect trials independent of criteria (i.e., metacognitive sensitivity; Maniscalco and Lau, 2012), metacognitive bias quantifying how liberal or conservative the criteria were, and gamma correlations with contrast levels to assess whether subjective reports differentiate between different levels of quality of stimulation. As both the presence as well as the absence of effects are relevant for the present research question, Bayes factors are used to test hypotheses, which provide a continuous measure of how the evidence supports the alternative hypothesis over the null hypothesis and vice versa (Rouder et al., 2009; Dienes, 2011).

## Materials and Methods

### Participants

Twenty participants (four male, all right handed) took part in the experiment. The age of the participants ranged between 20 and 30 years, with a mean age of 23.9. All participants reported normal or corrected-to-normal vision, no history of neuropsychological or psychiatric disorders and no psychoactive medication. Participants gave written informed consent and received either course credits or ¬ 8 per hour for participation. The study protocol was approved by the ethics committee of the Deutsche Gesellschaft für Psychologie.

### Apparatus and Stimuli

The stimuli were presented on a ViewSonic Graphics Series G90fB CRT monitor with 19 inch screen size and at a refresh rate of 80 Hz placed in a distance of approximately 60 cm in front of the participant, located in a sound-attenuated and electrically shielded cabin.

The experiment was conducted using a Dell Precision T3400 PC with Windows XP, MATLAB 7.2, and Psychtoolbox (Brainard, 1997; Pelli, 1997). The target stimulus was either a circle (diameter: 0.2° of visual angle) or a square (size: 0.2° × 0.2°) textured with a binary grating. The lighter and darker luminance of the grating varied randomly across trials, resulting in six different levels of luminance contrasts: 0% (uniform stimulus luminance at 38.2 cd/m^2^), 2.2% (39.0 vs. 37.3 cd/m^2^), 3.9% (39.5/36.5 cd/m^2^), 5.0% (40.0 vs. 36.2 cd/m^2^), 5.5% (40.2 vs. 36.0 cd/m^2^), and 6.9% (41.1 vs. 35.8 cd/m^2^). The orientation of the texture was either horizontal or vertical and randomly varied across trials. The target stimulus was always presented at fixation in front of a gray (35.5 cd/m^2^) background.

Participants responded to the orientation task by pressing “A” or “S” on the keyboard with their left hands and made subjective reports on a continuous visual analog scale on a Cyborg V1 joystick (Cyborg Gaming, UK). Joysticks were previously observed to be a decent method to record subjective measures (Rausch and Zehetleitner, 2014).

### Trial Structure

**FIGURE 1:**
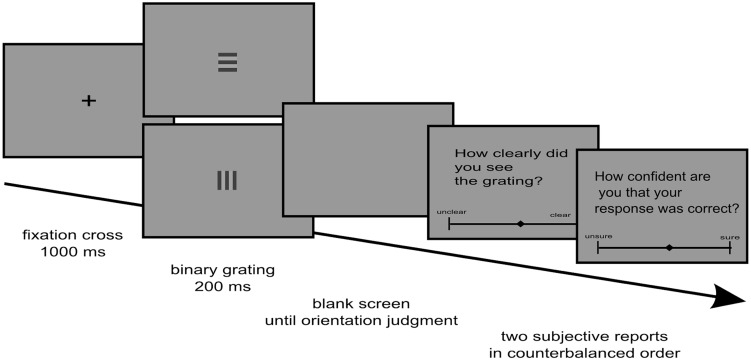
**Trial structure**.

As can be seen from **Figure 1**, each trial began with the presentation of a fixation cross at the screen center for 1,000 ms. Then, the target was presented for a fixed period of time of 200 ms, until it was replaced by a blank screen. The blank screen remained until participants had indicated whether the orientation of the grating had been horizontal or vertical. To prevent premature responses, there was a period of 600 ms after the stimulus when participants could not yet respond to the stimulus. Immediately afterward, two subjective scales were presented one after the other. For each subjective scale, a question was displayed on the screen, with a continuous rating scale presented underneath the question. For visibility, the question was “how clearly did you see the grating?” with the two anchors “unclear” and “clear.” For discrimination confidence, the question was “how confident are you that your response was correct?” with the anchors “unsure” and “sure.” Subjects always responded to both questions after each trial. The order of the two questions was counterbalanced across subjects. If the response to the task had been erroneous, “error” was displayed on the screen for 1,000 ms after the last subjective report, before the next trial started.

### Design and Procedure

Participants were instructed to report the orientation of the grating, their visual experience, and their confidence as accurately as possible without time pressure. The experiment consisted of a training block and nine experimental blocks of 42 trials each. In each block, every contrast value was presented seven times in random order. After each block, the percentage of errors was displayed to provide participants with feedback about their accuracy.

### Data Analysis

All analyses were conducted using the free software R 3.1.1. (R Core Team, 2014).

Metacognitive sensitivity, the degree to which subjective reports discriminate between correct and incorrect trials was quantified by meta-*d*_a_ (Maniscalco and Lau, 2012) using an implementation in the R (Rausch et al., 2015). Meta-*d*_a_ is expressed in units of discrimination sensitivity *d*_a_, allowing a direct comparison between meta-*d*_a_ and *d*_a_. The continuous subjective reports were discretized into 13 bins for the computation of meta-*d*_a_ (the same number of bins as in Rausch et al., 2015). Metacognitive bias was computed based on the meta-*d*_a_ algorithm as the average distance of rating criteria to the discrimination criterion on the decision axis assumed by the ideal observer signal detection model (Maniscalco and Lau, 2012). The correlation between subjective reports and stimulus contrast was assessed by ordinal Gamma correlation coefficients.

To analyze the probability of reporting visibility and confidence conditioned on the other report in the same trial without inflation of the number of conditions, subjective reports were dichotomized. As can be seen from Supplementary Figure 1, in trials when the stimulus was absent, most subjective reports fell below approximately 25% of the scale range, which we used as a cutoff to divide the continuous subjective reports. Visibility below 25% had been used as cutoff in previous studies as well (Del Cul et al., 2007).

Hypothesis testing was performed by Bayesian mixed linear regression models with default fixed-variance priors (Rouder and Morey, 2012) based on the R package Bayes Factor (Morey and Rouder, 2015). Separate models were fitted with average report, metacognitive sensitivity, metacognitive bias, and the correlation between reports and contrast as dependent variables and a random effect of participant on the intercept. Each model involved the predictors scale (visibility vs. confidence), and scale order (visibility first vs. confidence first); contrast and squared contrast centered to its mean were tested for average report, metacognitive sensitivity, and metacognitive bias; and trial accuracy (correct vs. incorrect) was included into the models predicting average reports and the correlation between reports and contrast. In addition, we also tested the interactions between scale and each of the other factors. To obtain Bayes factors for each effect, the marginal likelihoods of the model involving all effects was divided by the marginal likelihood of the model where the effect to be tested was dropped from the model. As priors, scaled inverse-chi-square priors on *g*-parameters were assumed (see Rouder and Morey, 2012, for the detailed model specification), using a scale parameter of 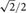 for discrete predictors (scale, scale order, accuracy), and 0.5 for continuous predictors (contrast, squared contrast). This choice of scale parameters reflects the equivalent of the default prior for the alternative hypothesis recommended for psychology (Rouder et al., 2009). As measure of effect size, we computed Δ*R*^2^ between the full model and the abridged model using the R libraries lme4 (Bates et al., 2014) and MuMIn (Nakagawa and Schielzeth, 2013; Barton, 2014). To compare meta-*d*_a_ and *d*_a,_ and to compare conditioned probabilities, a series of Bayesian *t*-tests based on a default Cauchy prior with a scale parameter of 1 was used (Rouder et al., 2009).

## Results

To facilitate reproduction of the present study and replication of its results (Ince et al., 2012; Morin et al., 2012; Simonsohn, 2013), the data and the analysis script were made available at the Open Science Framework^1^.

### Descriptive Statistics

Mean discrimination accuracy was 49.4% (*SEM* = 1.2) at the minimum contrast of 0 and 97.6% (*SEM* = 0.7) at the maximum contrast of 6.9%. Participants’ confidence in the correctness of the preceding discrimination response was 25.3% (*SEM* = 4.4) of the width of the visual analog scale at the minimum contrast and 92.4% (*SEM* = 2.5) at the maximum contrast. Likewise, participants reported an average degree of visibility of 13.6% (*SEM* = 3.6) at the contrast of 0 and 72.7% (*SEM* = 4.0) at the contrast of 6.9%.

### Average Reports

**FIGURE 2:**
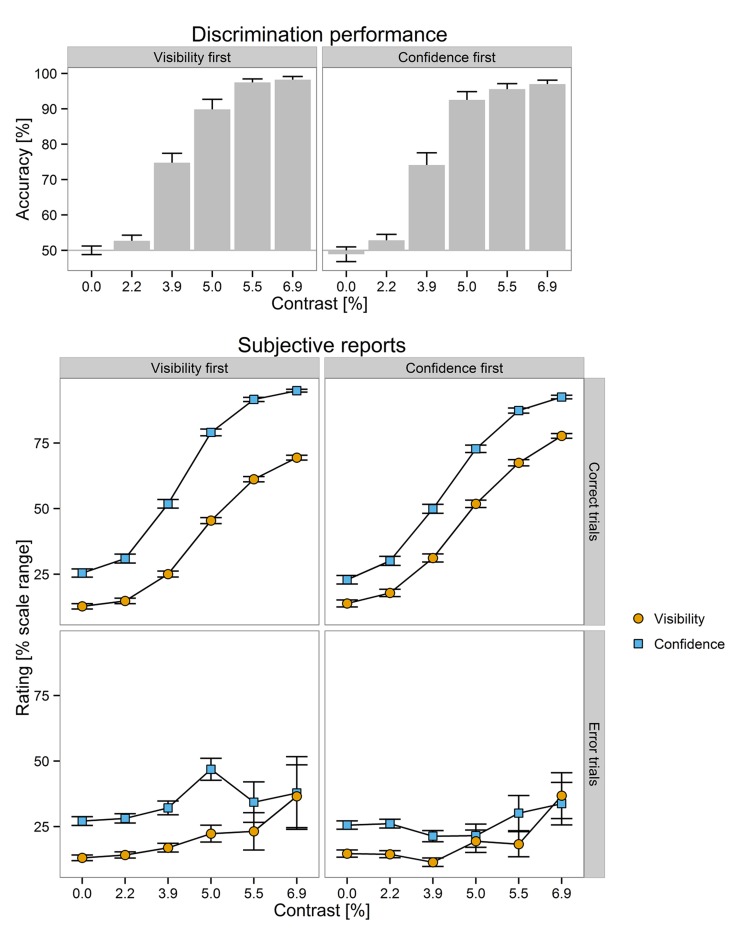
**(Upper)** Mean discrimination performance as a function of stimulus contrast and scale order (visibility first vs. confidence first, in separate columns). **(Lower)** Mean subjective reports as a function of trial accuracy (correct vs. incorrect trials, in separate rows), scale order (visibility first vs. confidence first, in separate columns), stimulus contrast (on the *x*-axis), and scale (visibility vs. confidence, in different colors). Errors bars ⇔ 1 *SEM*.

Average reports as a function of scale, scale order, and contrast can be seen in **Figure 2**. The data of single participants is found in Supplementary Figure 2. The Bayesian regression analysis indicated very strong evidence for effects of scale, BF_10_ = 2.4 × 10^14^, Δ*R*^2^ = 0.06, accuracy, BF_10_ = 4.5 × 10^31^, Δ*R*^2^ = 0.12, contrast as a linear predictor: BF_10_ = 1.2 × 10^56^, Δ*R*^2^ = 0.25, and contrast as a quadratic predictor: BF_10_ = 2.8 × 10^8^, Δ*R*^2^ = 0.03. The evidence for an interaction between scale and accuracy was not conclusive, BF_10_ = 2.3. For the other effects, the Bayes factor provided varying degrees of evidence for the absence of the effect: The support of the *H*_0_ was strong for the interactions between scale and linear contrast, BF_10_ = 0.098. The evidence for the *H*_0_ was positive for the effect of scale order, BF_10_ = 0.28, as well as for the interactions between scale and quadratic contrast, BF_10_ = 0.24. Finally, the evidence against an interaction between scale and scale order was not conclusive, BF_10_ = 0.40.

### Conditioned Probabilities

**FIGURE 3:**
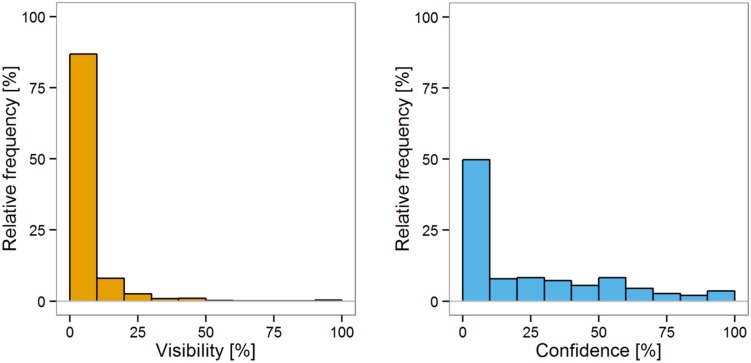
**Distribution of visibility **(Left)** and confidence **(Right)** in trials when the other subjective report in the same trial fell below 25% of the scale range**.

The distribution of subjective reports of visibility in trials when participants’ confidence was less than 25% of the scale range and the corresponding distribution of confidence in trials when visibility was below 25% are depicted in **Figure 3**. The distribution of visibility and confidence as a function of other ranges of the second subjective report can be found in Supplementary Figures 3 and 4. Importantly, in trials when confidence was below 25% of the scale, visibility was also below 25% in 96.2% of trials. In trials when visibility was below 25% of the scale, confidence fell in the same range in only 61.7%. A Bayes factor indicated there was strong evidence that the probabilities of confidence and visibility below 25% conditioned on the other subjective report falling below 25% were not the same, BF_10_ = 138.5. Likewise, when participants reported that their discrimination confidence was above 25% of the scale, participants reported that the visibility was at least 25% in 98.0% of trials. In contrast, when participants reported that their confidence was at least 25%, they also reported that their visibility fell in the same range in only in 75.9%. A Bayes factor indicated very strong evidence that the probabilities of a report above 25% conditioned on the other report above 25% were different, BF_10_ = 3.3 × 10^3^.

### Metacognitive Sensitivity

**FIGURE 4:**
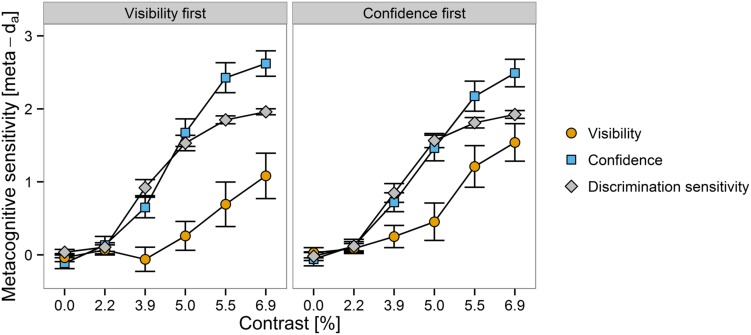
**Discrimination sensitivity and metacognitive sensitivity as a function of scale order (visibility first vs. confidence first, in separate panels) and contrast (on the *x*-axis).** Metacognitive sensitivity of visibility and confidence is depicted in different colors. Errors bars ⇔ 1 *SEM*.

Meta-*d*_a_ was smaller when subjective reports were about visibility, *M* = 0.5, *SEM* = 0.1, compared to meta-*d*_a_ computed from discrimination confidence, *M* = 1.2, *SEM* = 0.1, and compared to *d*_a_, *M* = 1.1, *SEM* = 0.04, which measures objective discrimination performance on the same scale as meta-*d*_a_. As can be seen from **Figure 4**, the effect of visibility vs. confidence on meta-*d*_a_ emerged already at the contrast of 1.6%. In addition, metacognitive sensitivity of both scales appeared to be slightly smaller when they preceded the other scale. The Bayesian regression analysis indicated very strong evidence for effects of scale, BF_10_ = 1.2 × 10^19^, Δ*R*^2^ = 0.12, contrast as a linear predictor, BF_10_ = 2.7 × 10^52^, Δ*R*^2^ = 0.47, contrast as a quadratic predictor: BF_10_ = 1.3 × 10^8^, Δ*R*^2^ = 0.05, as well as an interaction between scale and linear contrast, BF_10_ = 1.2 × 10^9^, Δ*R*^2^ = 0.05. In contrast, the Bayes factors indicated strongly that there was no interaction between scale and quadratic contrast, BF_10_ = 0.079, and provided some evidence that there was no effect of scale order, BF_10_ = 0.26. Finally, the evidence concerning an interaction between scale and scale order was not conclusive, BF_10_ = 2.8. To explore this potential interaction, we performed separate analyses for each order of subjective reports. Effects of scale were observed both when visibility preceded confidence, BF_10_ = 3.2 × 10^16^, and vice versa, BF_10_ = 4.2 × 10^6^. However, the magnitude of the effect appeared to be increased when participants reported visibility first, Δ*R*^2^ = 0.18, compared to Δ*R*^2^ = 0.07 with the opposite order.

A series of direct comparisons between metacognitive sensitivity as expressed in meta-*d*_a_ and the corresponding measure of discrimination performance *d*_a_ can be found in **Table 1**. We observed that meta-*d*_a_ as computed from visibility ratings was lower than what would be expected from *d*_a_ starting from a contrast level of 3.9%. In contrast, meta-*d*_a_ computed from confidence ratings was greater than *d*_a_ at contrast levels of 5.5 and 6.9%.

**Table 1:** Direct comparison between metacognitive sensitivity and discrimination performance.

Contrast level [%]	Discrimination performance *d*_a_		Visibility meta-*d*_a_			Confidence meta-*d*_a_		
	*M*	*SEM*	*M*	*SEM*	BF_10_	*M*	*SEM*	BF_10_
0.0	0.01	0.03	0.00	0.04	0.2	-0.09	0.06	0.3
2.2	0.11	0.04	0.08	0.05	0.2	0.13	0.07	0.2
3.9	0.88	0.08	0.10	0.11	774.4	0.68	0.09	1.4
5.0	1.55	0.07	0.36	0.16	8015.9	1.57	0.13	0.2
5.5	1.83	0.04	0.95	0.21	104.6	2.30	0.14	31.6
6.9	1.94	0.03	1.31	0.20	7.9	2.56	0.13	718.7

*Bayes factors compare *d*_a_ and meta-*d*_a._*

### Metacognitive Bias

**FIGURE 5:**
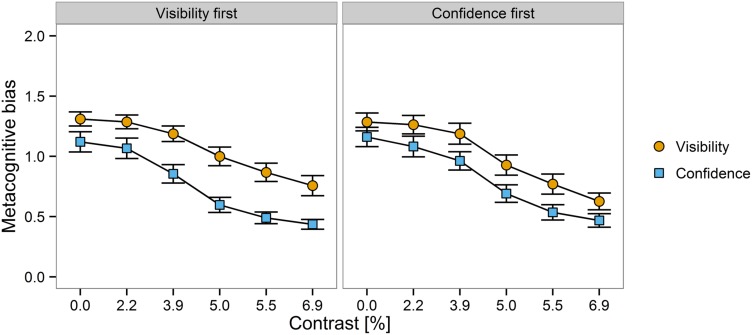
**Mean metacognitive bias as a function of scale order (visibility first vs. confidence first, in separate panels), contrast (on the *x*-axis), and scale (visibility vs. confidence, in different colors)**. Greater values indicate a more conservative reporting strategy. Errors bars ⇔ 1 *SEM*.

Participants were always more liberal in reporting their confidence in being correct in the discrimination task than in reporting their subjective visibility of the stimulus except trials where contrast was maximal and the response was incorrect. The estimated distance of second order criteria to the discrimination criterion was smaller when subjective reports were about discrimination confidence, *M* = 0.8, *SEM* = 0.4, compared to when reports where about visibility, *M* = 1.3, *SEM* = 0.5. The Bayesian regression analysis revealed very strong evidence for effects of scale, BF_10_ = 1.1 × 10^24^, Δ*R*^2^ = 0.12, and contrast, linear trend: BF_10_ = 8.2 × 10^57^, Δ*R*^2^ = 0.43, quadratic trend: 3.3 × 10^6^, Δ*R*^2^ = 0.03, and positive evidence for an interaction between scale and scale order, BF_10_ = 5.8, Δ*R*^2^ = 0.01. There was also substantial evidence against the effect of scale order, BF_10_ = 0.33, as well as against the interaction between scale and contrast, linear trend: BF_10_ = 0.16, quadratic trend: BF_10_ = 0.21. To resolve the interaction between scale and scale order, we performed separate analyses for each order of subjective reports. Effects of scale were observed both when visibility preceded confidence, BF_10_ = 3.2 × 10^16^, and vice versa, BF_10_ = 4.2 × 10^6^. However, the magnitude of the effect was increased when participants reported visibility first, Δ*R*^2^ = 0.18, compared to the opposite order, Δ*R*^2^ = 0.07 (see **Figure 5**).

### Correlation between Subjective Reports and Stimulus Contrast

**FIGURE 6:**
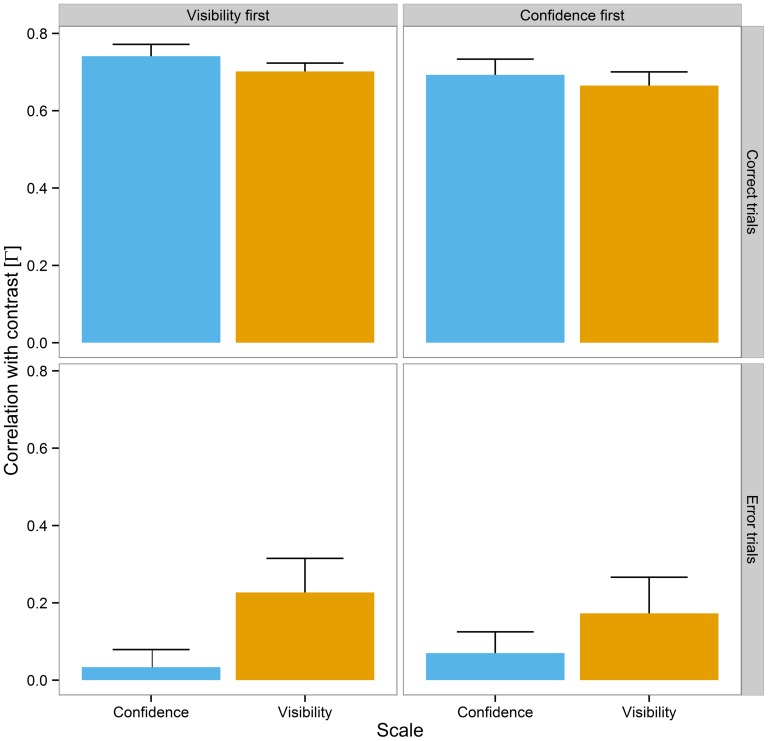
**Mean gamma correlation coefficients between subjective reports and contrast as a function of scale (visibility vs. confidence, in different colors), scale order (visibility first vs. confidence first, in separate columns), and trial accuracy (correct vs. incorrect trials, in separate rows).** Errors bars ⇔ 1 *SEM*.

As can be seen from **Figure 6**, the average gamma correlation coefficients between subjective reports and contrast was nearly the same when the discrimination response was correct, visibility: *M* = 0.68, *SEM* = 0.02, confidence: *M* = 0.72, *SEM* = 0.03. However, when the discrimination response was incorrect, visibility was still associated with contrast, *M* = 0.20, *SEM* = 0.06, while the correlation between confidence and contrast was close to being absent, *M* = 0.05, *SEM* = 0.03. The Bayesian regression analysis indicated very strong evidence for an effect of accuracy, BF_10_ = 1.0 × 10^25^, Δ*R*^2^ = 0.71, as well as positive evidence for an interaction between scale and accuracy, BF_10_ = 6.5, Δ*R*^2^ = 0.02. Moreover, the Bayes factors suggested no effect of scale order, BF_10_ = 0.31, and no interaction between scale and scale order, BF_10_ = 0.27. Finally, the evidence was not conclusive for the effect of scale, BF_10_ = 0.62. To examine the interaction between scale and accuracy, we performed separate analyses for correct and incorrect trials. While there was positive evidence for an effect of scale when trials were incorrect, BF_10_ = 3.9, Δ*R*^2^ = 0.09, the evidence was not conclusive when the trial was correct, BF_10_ = 2.0.

## Discussion

The present study was conducted to investigate subjective reports of visibility and discrimination confidence during a low-contrast discrimination task. We observed that participants reported to be confident about the discrimination response being correct although they reported no visibility of the stimulus from time to time, but they hardly ever reported visibility in absence of discrimination confidence. Moreover, discrimination confidence was more efficient in predicting trial accuracy than visibility was. The analysis of metacognitive bias suggested that participants applied more conservative criteria for visibility compared to discrimination confidence. Finally, both visibility and confidence were associated with contrast in correct trials. However, only visibility was positively related to stimulus contrast in erroneous trials, indicating that participants were able to differentiate between lower and higher stimulus contrasts even in trials when they were not able to differentiate between correct and incorrect orientations. These effects of visibility vs. discrimination confidence were qualitatively the same no matter which report was made first; however, the effect on metacognitive bias was increased when visibility was reported before confidence.

### Should We Trust Participants about Feelings of Confidence at No Visibility?

The pattern of participants reporting confidence in being correct without visibility observed in the present paradigm raises the question whether participants “really” experience confidence without visual experiences, or whether they only report confidence more liberally than visibility. For blindsight type 2, it was argued that many blindsight patients experience some residual visual phenomenology despite their reports of being blind (Foley, 2014). In particular, under certain circumstances, some blindsight patients have reported to experience visual conscious contents (Overgaard et al., 2008; Ffytche and Zeki, 2011). As first-person experiences can never be observed from the third-person perspective (Nagel, 1974; Jackson, 1982), nobody can conclusively rule out participants having visual experiences without reporting them. Nevertheless, it seems rather odd why participants would readily acknowledge feelings of rightness while being reluctant to admit visual contents. Moreover, the present pattern of confidence without visibility seems to be reasonably robust as it was replicated in patients (Sahraie et al., 1998; Carota and Calabrese, 2013) and healthy human observers in a range of tasks (Schlagbauer et al., 2012; Charles et al., 2013; Zehetleitner and Rausch, 2013). Finally, the analysis of metacognitive sensitivity suggests that the effect of confidence and visibility as contents of subjective reports is not exhausted by criterion setting. While future research on the relation of sensory and non-sensory conscious contents is clearly important, it seems appropriate to start reflecting upon the implications of such a distinction.

### Implications for Theories of Consciousness

Is the dissociation between subjective reports of visibility and discrimination confidence consistent with the existing theories of consciousness? In the following, we discuss whether such a distinction can be integrated into three of most influential concepts of conscious awareness for contemporary research:

(i) Global workspace theory (Dehaene and Naccache, 2001; Baars, 2002, 2005)
(ii) Higher-order theory (Carruthers, 2011; Lau and Rosenthal, 2011; Timmermans et al., 2012)
(iii) Phenomenal consciousness (Block, 2002).

Can global workspace theory account for distinction between visibility and confidence? Global workspace theory assumes that representations encapsulated in specialized brain systems are unconscious. These representations can be made available to multiple brain systems via the global workspace. The global availability of representations through the workspace is subjectively experienced as a conscious state (Dehaene and Naccache, 2001; Baars, 2002). An important tenet of global workspace theory is that conscious access is all-or-nothing (Dehaene et al., 2003). Conscious access depends on a cerebral “ignition” where neural activity spreads from sensory cortical areas to frontal and parietal areas, making perceptual contents available to multiple cognitive functions. If global ignition does not occur, perceptual contents are not available for report (Dehaene et al., 2006). How can the binary characteristic of the global workspace be reconciled with the intermediate state where participants report some confidence in being correct in the discrimination task about the stimulus, but report no visibility of the stimulus?

One possibility to explain the visibility/confidence distinction within the framework of the global workspace is in terms of the partial awareness hypothesis (Kouider et al., 2010). It assumes that a stimulus is represented by a hierarchy of features, with low-level features at the bottom and increasingly complex features at the top. Separate features can be consciously accessed independently from the other features. Partial awareness is a state where some of the features of a stimulus are consciously accessible but others features are missing. If participants are in a state of partial awareness, conscious access to the task-relevant stimulus feature may be sufficient for reporting confidence about the discrimination response (Dienes, 2004, 2008). Moreover, a state of partial awareness may be insufficient to create a coherent visual working memory representation which may be strongly involved in subjective reports of visibility. Conscious visual working memory representations are not dependent on feature-specific mechanisms (Bona et al., 2013). Alternatively, a report about visibility may require conscious access to a greater number of stimulus features (Rausch et al., 2015). After all, a striking feature of consciousness is the so-called unity of experiences: Humans do not experience color, shape, location, etc., of a stimulus as separate; instead, the conscious experience of the separate features of a stimulus seems to be integrated to one visual object (Bayne and Chalmers, 2003). The partial awareness hypothesis elegantly explains further details of the present results: If task-irrelevant stimulus features contribute only to subjective reports of visibility, they would not as efficient in predicting trial accuracy as confidence ratings. In addition, it explains why visibility is associated with contrast in incorrect trials, because access to task-irrelevant features is more likely at higher contrast even when the task-relevant feature is not available.

A second explanation is based on unconscious evidence accumulation. According to this theory, subliminal stimuli possess sufficient energy to evoke a wave of activity in feed-forward wave of activation in specialized processors, but insufficient energy to trigger global neural activity necessary for reports of visibility (Dehaene et al., 2006; Dehaene, 2010). This unconscious feedforward sweep may even reach higher areas, thereby leading to above chance performance as well as error detection in the absence of consciousness (Charles et al., 2013, 2014). If confidence can be generated by the unconscious feedforward sweep, it be explained why the condition to report confidence is more frequently fulfilled than the condition to report visibility. However, one aspect of the data fits slightly better to the partial awareness hypothesis: If dissociations between visibility and confidence were only due to unconscious feedforward processing, the maximal difference between visibility and confidence would be expected at intermediate contrast levels. At lower contrasts, feedforward processing would often be not sufficiently strong to reach higher areas, and consequently participants would report no confidence and no visibility. At higher contrasts, the processing would be strong enough to trigger a global workspace most of the time, and participants would consequently report confidence as well as visibility. A maximal discrepancy between visibility and confidence at medium contrast levels predicts an interaction effect in the regression analysis between scale and a quadratic effect of contrast. Squared centered contrast is maximal when contrast is either lowest or strongest, and minimal at intermediate contrast levels. Consequently, the interaction effect between scale and quadratic contrast would be strong if the effects of scale were the same at lowest and strongest contrast levels, and if the effects of scale at the extreme contrast levels were different from the effect of scale at intermediate contrast levels. In the present data, there was always evidence against such an interaction, indicating that unconscious evidence accumulation does not explain all characteristics of the difference between visibility and confidence.

According to higher-order theories of consciousness, a mental state of an observer is conscious if the mental state is accompanied by a higher-order mental state that represents the observer as being in a particular mental state (Carruthers, 2011; Lau and Rosenthal, 2011; Timmermans et al., 2012). According to different flavors of higher-order consciousness, this higher order mental state can be a thought (Rosenthal, 1986), a percept (Lycan, 2004), or a statistical inference (Lau, 2008a). In the framework of higher-order theories, subjective reports about visibility indicate whether participants possess a higher order mental state about seeing the stimulus, while discrimination confidence demonstrate higher order mental states about discrimination performance. Consequently, the present data suggests that there can be higher-order states about discrimination performance without higher-order states about perception: Participants know that they have responded correctly, but they do not know that they have seen the stimulus.

Are higher-order states about discrimination performance without higher-order states perception consistent with higher order theories? At first glance, it may seem they are not: Higher order theories strongly emphasize the link between consciousness and metacognition (Rosenthal, 2000). Under the assumption that subjective reports of visibility do not depend on metacognition, confidence in task accuracy without visibility of the stimulus undermines the strong link between consciousness and metacognition and thus one of the core tenets of higher-order theories (Charles et al., 2013, 2014). However, the assumption is controversial: Both subjective reports about visibility and confidence require metacognitive processes with the only difference that subjective measures about visibility require metacognition of visual perception, not discrimination performance (Zehetleitner and Rausch, 2013). Consistent with this, subjective reports about visibility are associated with neural activity in dorsolateral prefrontal cortex (dlPFC) as suggested by fMRI (Lau and Passingham, 2006) and theta-burst TMS studies (Rounis et al., 2010). The dlPFC is a brain region closely related to metacognition (Fleming et al., 2010; Fleming and Dolan, 2012). Overall, the proposition that confidence in absence of visibility indicates consciousness without metacognition appears rather difficult to defend.

What mechanism can account for the occurrence of higher-order states about discrimination performance without higher-order states about perception? The first two proposals both assume that confidence requires additional metacognitive processes. First, the metacognitive process specific to confidence may be an unconscious error monitoring system (Charles et al., 2013, 2014). This error monitoring process could be informed by perceptual processes too weak to trigger higher-order thoughts about perception, thus explaining why participants report confidence in being correct but do not report a visibility of the stimulus. In line with this view, metacognitive sensitivity calculated from confidence was greater than expected from discrimination sensitivity at higher contrasts, which may suggest that error monitoring processes may be involved in discrimination confidence. It is possible that error detection contributes to visibility as well (cf. Wierzchoń et al., 2014), but this contribution could be modest or overlaid by noise, which is why metacognitive sensitivity of visibility increases only moderately at higher contrasts, and remains smaller than expected from discrimination performance. Nevertheless, the present data is fully compatible with error monitoring exclusively involved in confidence.

The second proposal suggests that subjective reports about visibility are generated by a simple metacognitive process of monitoring one’s experience. Confidence is thought to stem from a more complex metacognitive process that relates the output of the first metacognitive process to one’s accuracy in the task (Overgaard and Sandberg, 2012). This theory predicts that higher order thoughts about discrimination performance are conditioned on higher-order thoughts about perception of the stimulus. While this view was developed to explain the data of a previous study (Sandberg et al., 2010), in the present data, confidence is not conditioned on visibility; in fact, the criterion of discrimination confidence is lower, not higher than the criterion of visibility. In addition, metacognitive sensitivity of confidence was greater than of visibility, suggesting that confidence does not require a more complex judgment than visibility does.

According to a third proposal, many participants may engage in mental imagery to visualize the appearance of the stimulus in order report visibility. The ability of mental imagery is reduced by dynamic visual noise (Bona et al., 2013). If discrimination confidence requires less mental imagery than visibility and if there was noise in the system, mental imagery could explain why participants acquired more frequently and more precise higher order states about discrimination performance than higher-order states about perception, explaining both the difference in criteria and metacognitive sensitivity. Notably, none of these three proposals explains the pattern of correlation with stimulus contrast.

The final possibility is a variant of the partial awareness hypothesis framed within a higher order framework (see above): Partial awareness is a state where some features of the stimulus are globally accessible while others remain inaccessible (Kouider et al., 2010). Global access allows a great variety of cognitive systems to make use of the perceptual information (Dehaene and Naccache, 2001; Baars, 2002); one of the cognitive functions enabled by global access could be metacognition. Consequently, in a state of partial awareness where the task-relevant feature is accessible, participants may be able to form a higher order thought about discrimination performance and thus report being confident. However, other features of the stimulus may be inaccessible and so participants lack a higher order thought about perception and report no visibility accordingly. Such a higher-order framing of the partial awareness hypothesis has the same explanatory power as the original partial awareness hypothesis with respect to criteria, metacognitive sensitivities, and correlations with stimulus contrast.

Finally, is confidence without visibility consistent with the theory of phenomenal consciousness (Block, 2002)? Conscious awareness in the sense of phenomenal consciousness is defined as what-it-is-like to have an experience of an external stimulus or inner events (Nagel, 1974; Chalmers, 1994; Block, 2002). It is often critically discussed whether it makes sense to interpret participants’ subjective reports as evidence for participants’ private subjective experience (Dennett, 2007; Cohen and Dennett, 2011). To explore potential implications of our data, let us assume for now that when participants report a specific experience, it can be reasonably assumed that another human being would experience something similar in the same situation (see, e.g., Velmans, 2000, 2007). What could be the experience underlying participants’ reports of confidence when they do not report a visible stimulus? One possibility is that the phenomenology of participants that report discrimination confidence but no visual experience is rightness, the core feeling of positive evaluation, coherence, and meaningfulness (Mangan, 2001). Another possibility is the also non-sensory feeling-of-knowing: Participants have the experience that they know what the stimulus feature is, but the stimulus does not create visual phenomenology. Feeling-of-knowing has been originally described in the context of metamemory (Nelson and Narens, 1990; Koriat, 2007), but subliminal perception may be able to generate feelings-of-knowing as well (Mangan, 2001). The final possibility is a state similar to blindsight type 2: Their residual phenomenology is characterized by the awareness of the event, but without the phenomenology of normal seeing (Zeki and Ffytche, 1998; Sahraie et al., 2002). Normal observers may have a similar experience if the stimuli are just at the threshold of conscious perception (Ramsøy and Overgaard, 2004).

There is one specific thesis about phenomenal consciousness that at first glance appears to be at odds with the current data: According to the overflow hypothesis, the contents of short-term sensory buffers are associated with phenomenal experience (Block, 2011). This short-term sensory buffer stores all visual objects for a short period of time, until it is overridden by the next stimulation. However, participants are only able to make correct discrimination judgments about 4 ± 1 objects, as cognitive access to the contents of sensory buffers is limited by the capacity of working memory (Sligte et al., 2008; Vandenbroucke et al., 2011). As the capacity of the conscious sensory buffer is much larger than the capacity of working memory, the overflow hypothesis explains why participants are only able to make correct task responses about a small number of display items, although they report an experience of a rich phenomenal world (Block, 2011).

The standard and widely debated case is that phenomenal consciousness exceeds cognitive access. In present data, the relation between visual consciousness and access appears to be the other way round: Participants report to be confident more often than they report visibility. Conscious access is a requirement for all subjective measures, as they require that neural systems engaged in decision making and language need to receive inputs from perceptual processes. However, although participants had conscious access when they reported they felt confident about task response, they reported no conscious visual experience. A similar pattern was reported from a patient suffering from achromatopsia (Carota and Calabrese, 2013): After bilateral temporal–occipital lesions, that patient reports to be color-blind although he performs accurately in a color recognition task. A potential explanation why the relation between phenomenal consciousness and conscious access varies is the number of items in the display: Visual short-term memory always used arrays of multiple stimuli. In contrast, the present studies always presented one stimulus at the screen at fixation. Consequently, phenomenal consciousness may overflow cognitive access only for stimuli outside of the focus of attention, while at the focus of attention, conscious access occurs more frequently than phenomenal experience.

### Implications for Mathematical Models of Subjective Reports

The present data may also be informative for mathematical models of subjective reports. Many key results can be explained in terms of signal detection theory (SDT). In particular, the analysis of metacognitive bias suggested that discrimination confidence is associated with rating criteria closer to the discrimination criterion. Likewise, the lower metacognitive sensitivity of visibility compared to confidence may be formally described by criterion jitter (Ko and Lau, 2012) or by decreasing signal (Barrett et al., 2013).

However, there are two aspects of the present data may posit a challenge to SDT and other models of discrimination confidence: First, many models imply that there is a positive correlation between confidence and stimulus contrast in correct trials and a negative correlation between confidence and stimulus contrast in incorrect trials (Kepecs et al., 2008; Moran et al., 2015). However, there was no association between confidence and contrast in incorrect trials and the relation between visibility and contrast was just reversed to what would be expected from standard SDT. Moreover, metacognitive bias decreased with increasing stimulus contrast, suggesting that participants lowered their criteria when stimulus quality was high. As stimulus contrast varied randomly from trial to trial, participants lowered their criteria based on their percept of the stimulus in the current trial. A possible explanation for this pattern of results is that participants might not exclusively consider the evidence when making a subjective report, but at least subjective reports of visibility may reflect heuristic computations of the magnitude of sensory data (Aitchison et al., 2015). Nevertheless, our paradigm was not designed to test SDT models; in particular, as both visibility and confidence reports were placed within single trials, it is possible that the absence of a negative correlation between stimulus contrast and discrimination confidence in incorrect trials is due to an interaction between confidence and the visibility rating. Future studies appear necessary to substantiate this result.

Second, metacognitive sensitivity calculated from discrimination confidence was greater than expected from discrimination performance at higher contrasts, indicating that subjective reports of confidence were based on more evidence than discrimination responses (Barrett et al., 2013). A potential explanation would be that evidence continues to be accumulated even after the discrimination response (Pleskac and Busemeyer, 2010; Moran et al., 2015; Murphy et al., 2015).

### Implications for Measuring Conscious Awareness

It is widely assumed in the field of consciousness research that subjective reports of visibility and discrimination confidence are equally valid, and thus typically used and/or interpreted as interchangeable (Seth et al., 2008; Lau and Rosenthal, 2011; Ko and Lau, 2012). As subjective reports of visibility and discrimination confidence are associated with different criteria and metacognitive sensitivities, the present experiment implies in accordance with some previous studies that subjective reports of visibility and confidence should not be considered *a priori* as equivalent measures of conscious awareness (Charles et al., 2013; Zehetleitner and Rausch, 2013; Rausch et al., 2015).

This is relevant for a series of studies that compared different subjective measures with the aim to identify appropriate scales to measure conscious awareness (Dienes and Seth, 2010; Sandberg et al., 2010, 2011; Wierzchoń et al., 2012, 2014; Szczepanowski et al., 2013; Rausch and Zehetleitner, 2014). This research program rests on the assumption that the scales under comparison are equally valid from a conceptual point of view, but some of these scales have better empirical properties than others. For example, some scales may correlate more strongly with task performance indicating that these are more sensitive measures of awareness (Overgaard and Sandberg, 2012). Alternatively, all scales may be sensitive to the same continuum of awareness, but different scale measure different ranges of the unawareness-awareness spectrum (Wierzchoń et al., 2012).

In contrast to the assumption of equal validity of confidence and visibility, we suggest whenever experiments suggest visibility and confidence do not converge to the same results, researchers need to consider carefully which conscious contents are relevant for their specific research question, and choose the content of their subjective measure accordingly: Some studies investigate the visual experience of a specific stimulus feature, e.g., studies investigating the neural correlate of experiencing “red” when seeing a red apple. In this case, participants should report their conscious experience of this particular feature. If participants were asked about their confidence in a task instead, there would be a risk that participants had just an intuition of being correct without visual experience, resulting in false positives (Rausch et al., 2015). In contrast, if a study is about all conscious contents underlying performance in a specific task, participants should report their confidence in being correct as subjective reports of visibility may lead to misses in this case (Dienes, 2004, 2008). If all conscious contents are relevant, researchers may want to consider if it is feasible to use several measures with different contents.

However, it is an open empirical question what is the set of experimental paradigms where discrimination confidence and visibility dissociate. The present paradigm differed from standard studies of implicit cognition in several aspects: Participants received feedback, two subjective reports were collected per single trial, the scale minima were labeled as “unsure” or “unclear” instead of “guessing” or “unseen,” and the reports were collected using visual analog scales and joysticks. Of course, the suitability of scales for studying implicit cognition is tested more rigorously when the paradigm is representative for the kind of experiments where subjective measures are commonly used. When discrimination confidence was compared to the PAS in more standard no-feedback, between-subject designs, the PAS was associated with greater metacognitive sensitivity than discrimination confidence (Sandberg et al., 2010; Wierzchoń et al., 2014). However, the PAS was designed to measure both visual contents as well as several non-sensory experiences and is consequently not a measure of visual experience only. Future studies appear necessary to investigate whether the effect of confidence vs. visibility as content of subjective measures on criteria and metacognitive sensitivity generalizes to between-subject designs without feedback, but with one report per trial, four category scales and standard scale labels.

## Conclusion

The effect of visual experience and discrimination performance as content of subjective reports on criteria and metacognitive sensitivity generalizes to a low contrast orientation task. We argue that the effect is consistent with the partial awareness hypothesis as well as higher-order theories of consciousness, but the observed pattern is just reversed to the overflow hypothesis of phenomenal consciousness.

## Author Contributions

MR and MZ conceived the research question, MR analyzed the data, MR and MZ interpreted the data and co-wrote the article.

## Conflict of Interest Statement

The authors declare that the research was conducted in the absence of any commercial or financial relationships that could be construed as a potential conflict of interest.

## Footnotes

1 https://osf.io/vk6fe/
