# Supplementary material for: Visibility Is Not Equivalent to Confidence in a Low Contrast Orientation Discrimination Task
Source: Front Psychol. 2016 Apr 26;7:591. doi: 10.3389/fpsyg.2016.00591 (PMC4874366; doi:10.3389/fpsyg.2016.00591)
Supplement: Supplementary file 1 [file Data_Sheet_1.PDF]

Supplementary Material

Visibility is not equivalent to confidence in a low contrast orientation discrimination task

Manuel Rausch*, Michael Zehetleitner

*** * Correspondence:** Manuel Rausch, Catholic University of Eichstätt-Ingolstadt, Psychology II, Ostenstraße 25, 85072 Eichstätt, Email: manuel.rausch@ku.de

Supplementary Figures

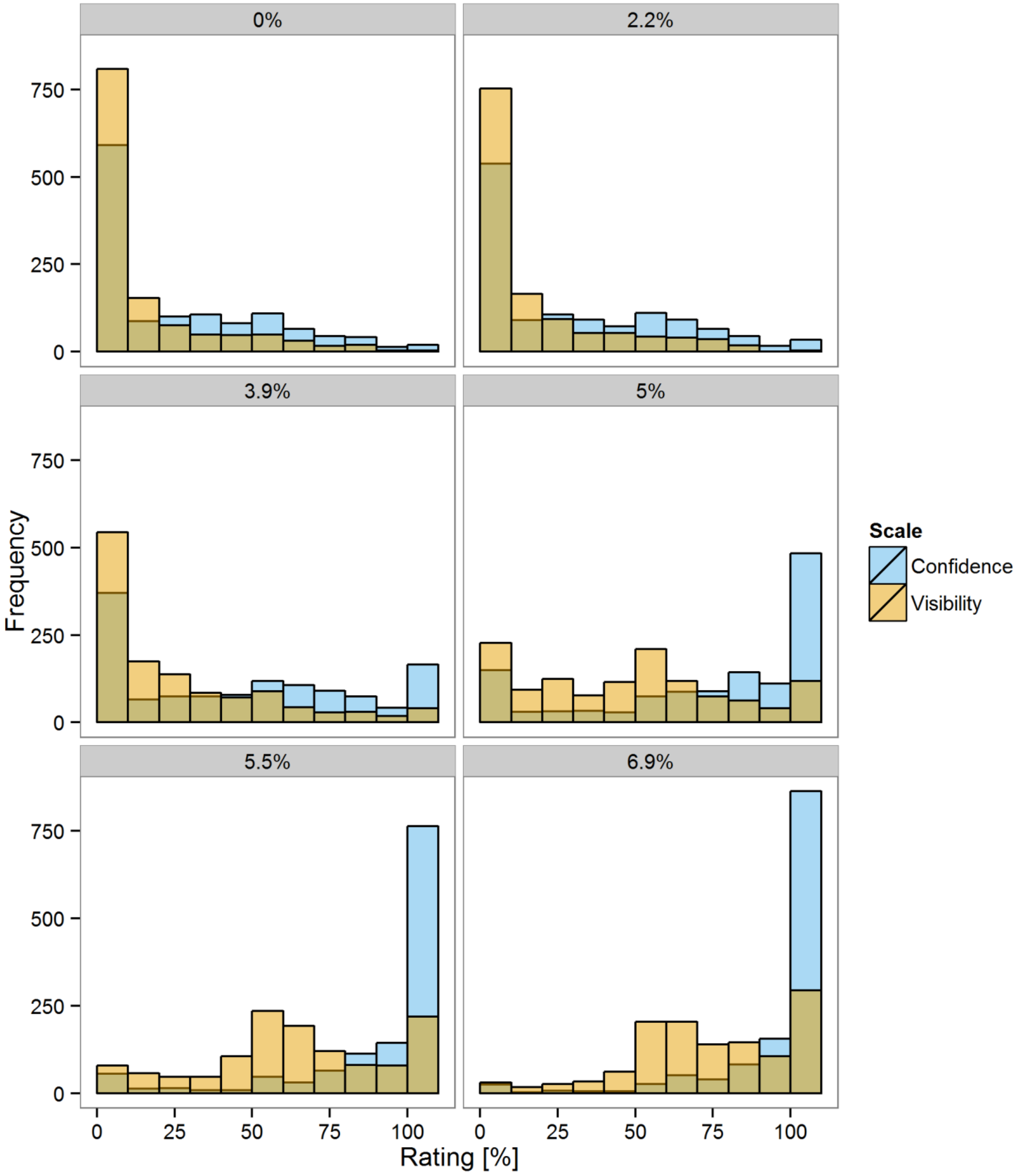

Supplementary Figure 1. Distributions of subjective reports of visibility and confidence as a function of contrast in separate panels.

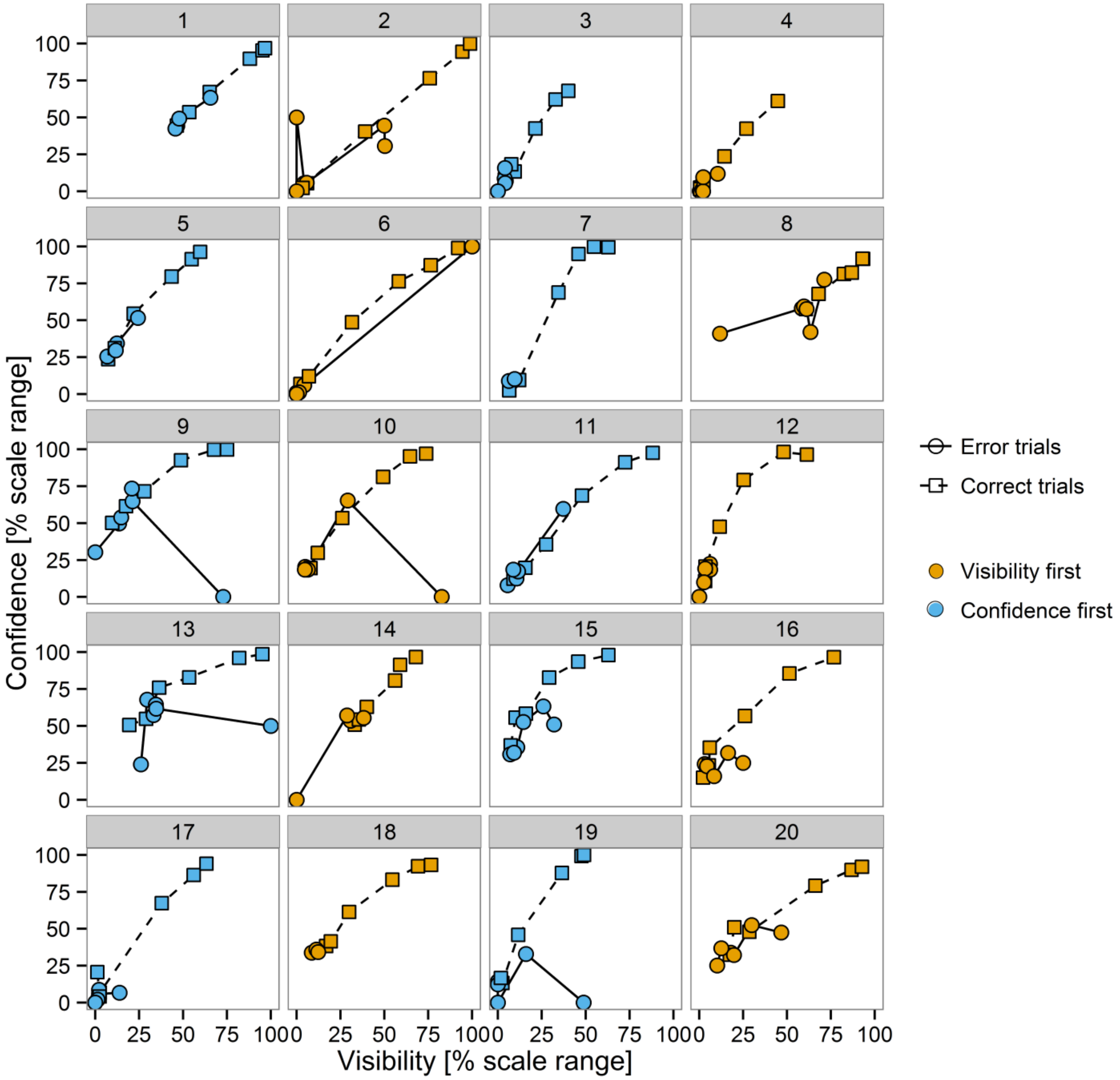

Supplementary Figure 2. The relation between visibility and confidence on the single-subject level. Each data point depicts mean visibility and mean confidence at one level of contrast. Orange symbols indicate participants who reported visibility first, and confidence afterwards; blue symbols indicate participants who made these reports in the opposite order. Square symbols indicate correct trials, circles incorrect trials.

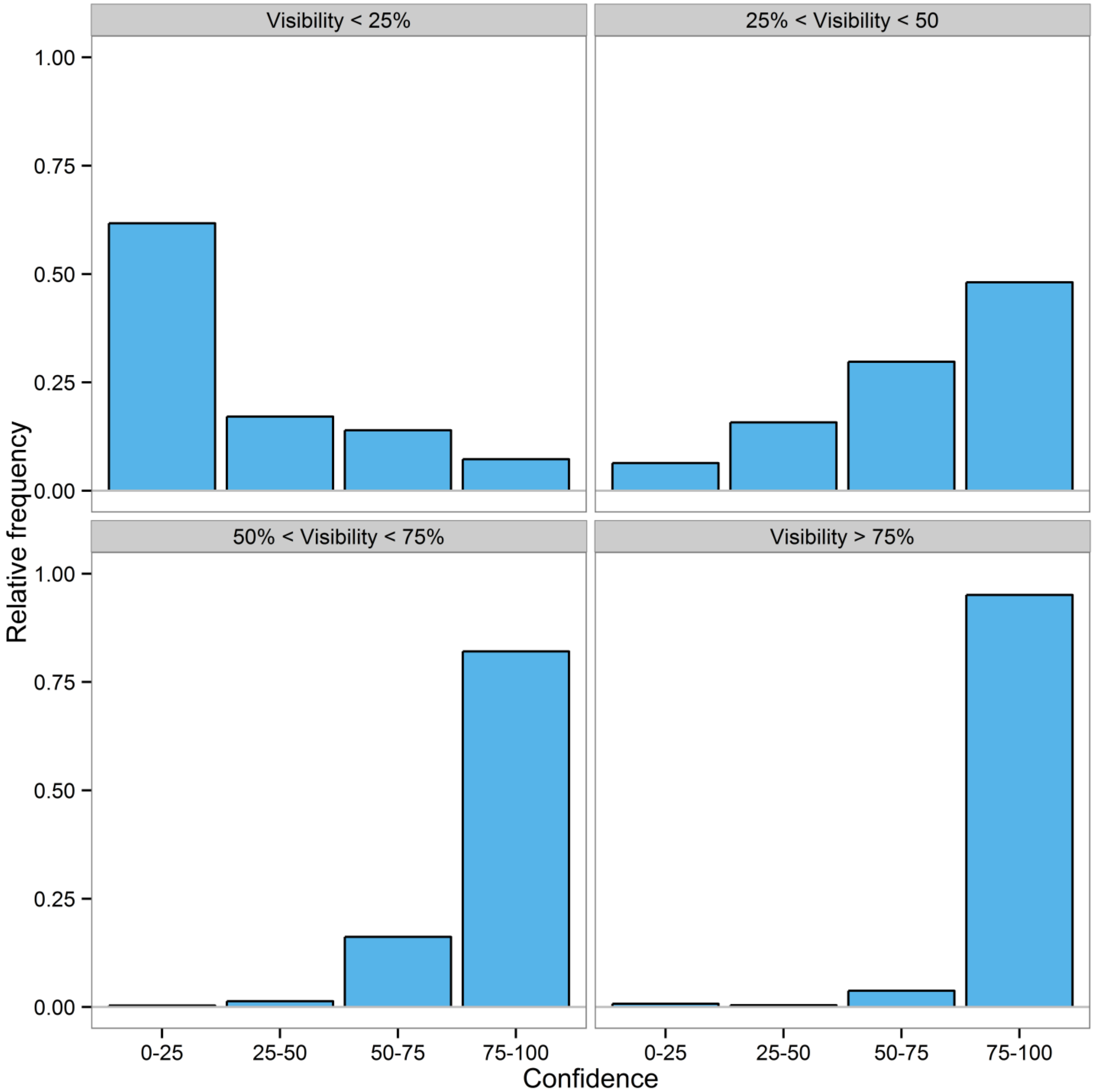

Supplementary Figure 3. Relative of confidence reports conditioned on visibility reported in the same trial.

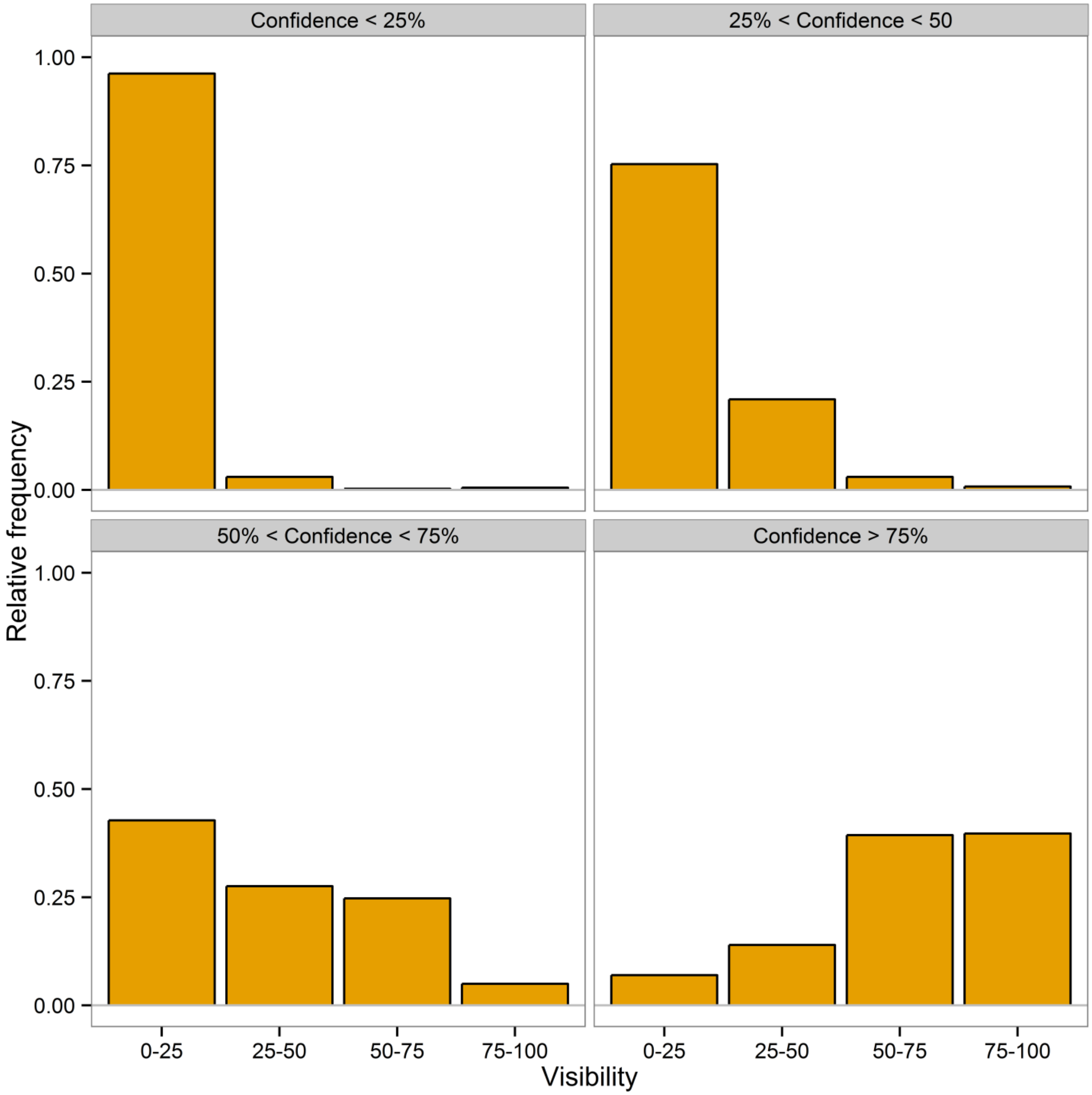

Supplementary Figure 4. Relative frequency of visibility reports conditioned on confidence reported in the same trial.
